# Hydrogen-centric machine learning approach for analyzing properties of tricyclic anti-depressant drugs

**DOI:** 10.3389/fchem.2025.1603948

**Published:** 2025-06-03

**Authors:** Simran Kour, J. Ravi Sankar

**Affiliations:** Department of Mathematics, School of Advanced Sciences, Vellore Institute of Technology, Vellore, Tamil Nadu, India

**Keywords:** tricyclic anti-depressant drugs, topological indices, QSPR, linear regression, support vector regression

## Abstract

**Introduction:**

Tricyclic anti-depressant (TCA) drugs are widely used to treat depression, but traditional methods for evaluating their physicochemical properties can be time-consuming and costly. This study examines how topological indices can help to predict the properties of TCA drugs, with a special focus on the role of the hydrogen representation.

**Methods:**

Two molecular configurations were analyzed: one with only explicit hydrogen and the other including all hydrogen atoms. To assess predictive performance, linear regression (LR) and support vector regression (SVR) models were employed.

**Results:**

The results showed that adding all hydrogen atoms showed strong correlations, especially for polarizability, molar refractivity, and molar volume. Among the models employed, SVR provided more accurate results. Additionally, hydrogen representation had a stronger impact on SVR's predictions.

**Discussion:**

These findings highlight the potential of using machine learning techniques in quantitative structure-property relationship (QSPR) models for more efficient and reliable predictions of drug properties.

## 1 Introduction

Mental health disorders are a group of psychiatric conditions that can severely impact an individual’s ability to function in everyday environment, resulting in difficulties with daily activities, social connections, and behavioral stability (Ejima et al., 2024). Conditions such as anxiety, addiction, depression, and bipolar disorder are common, with depression being a particularly pressing public health concern that demands effective treatment options (Kessler et al., 2007). TCAs rank among the most commonly prescribed medications for depression, with over 25 million prescriptions written annually in the United States. However, despite their effectiveness, TCAs are frequently linked in overdose incidents, with studies showing that they contribute to nearly 25% of overdose-related hospital admissions at a major medical center (Marshall and Forker, 1982; Vandel et al., 1997). According to the 2023 NSDUH Report, 22.8% of adults (58.7 million) experienced any mental illness (AMI) in the past year, and 4.5 million adolescents reported a major depressive episode, with 20% also experiencing substance use disorders. Suicide remains a major worry, with 5.0% of adults having serious thoughts about it, 1.4% making plans, and 0.6% attempting suicide (Health and Services, 2023). These concerning statistics highlight the critical importance of prioritising mental health rehabilitation and preservation. While laboratory-based drug development has played a key role in advancing treatments for mental health, it is often resource intensive (Insel et al., 2013). Computational modeling and predictive techniques offer promising alternatives that are both cost-effective and resourceful. These approaches not only enhance traditional drug discovery but also provide accessible and effective therapy for those with neuropsychiatric disorders.

The process of drug design and discovery is a complex, time-consuming, and costly. To optimize this process, researchers have increasingly turned to predictive modeling techniques, particularly in resource-limited scenarios or during medical emergencies. One such approach is QSPR modeling, which predict a drug’s physicochemical properties based on its molecular structure and descriptors, commonly referred to as topological indices. Chemical graph theory applies the principles of graph theory to chemistry by representing molecules as graphs, where vertices correspond to atoms and edges to chemical bonds (Thapar et al., 2022). Topological indices, numerical descriptors derived from these graphs, capture critical structural information (Gutman and Polansky, 2012; Gutman, 2006). These indices serve as essential tools in QSPR modeling, as they establish mathematical relationships between molecular structures and biological or physicochemical properties, particularly in pharmaceutical research (Abubakar et al., 2024a). One of the earliest and most well-known topological indices is the Wiener Index, introduced in 1947, originally designed to predict the physical properties of paraffin compounds (Wiener, 1947). In recent years, topological indices have gained widespread popularity in QSPR studies, offering a cost-effective and time-saving alternative to experimental methods. By enabling researchers to predict key drug properties, identify influential structural features, and optimize drug candidates, these indices play a crucial role in accelerating drug development and reducing reliance on expensive laboratory experiments (Parveen et al., 2022; Zaman et al., 2023).

The use of topological indices in pharmaceutical research has significantly increased in recent years, particularly in QSPR studies. But, most QSPR modeling studies primarily focused on classical graph-based topological indices and simple regression models to establish relationships between topological indices and the physical properties of compounds. A topological index that exhibits a strong linear correlation with a physical property is regarded as an effective descriptor for predicting that property (Zaman et al., 2024; Das et al., 2024; Arockiaraj et al., 2024; Huang et al., 2024; Hasani and Ghods, 2024). However, when the relationship between topological indices and physical properties is non-linear, more advanced approaches like machine learning, are employed to capture complex patterns and improve predictive accuracy (Fernández-Blanco et al., 2013; Madugula et al., 2021; Abubakar et al., 2024b). Zabidi et al. (2021) applied machine learning to predict HOMO and LUMO, minimizing the need for computationally expensive DFT calculations. Degree-based topological indices were employed in QSPR analysis to establish correlations with these properties and identified Linear Regression with Moment Balaban Indices as the most accurate model. Costa et al. (2020) proposed explored a novel method for QSAR and QSPR modeling through Molecular Graph Theory, emphasizing molecular fragment contributions. By combining Molecular Graph Theory, SMILES notation, and connection table data, they established an efficient method for fragment identification. Machine learning techniques produced accurate predictive models, and the study introduced Charming QSAR and QSPR, a Python tool designed for property estimation in chemical compounds. Abubakar et al. (2024a) analyzed neighborhood degree-based topological indices for QSPR modeling of anti-tuberculosis drugs, employing Support Vector Regression (SVR) and comparing it to linear regression. The results demonstrated that SVR as a better predictive tool, enhancing the understanding of the non-linear relationship. Author A and others applied QSPR modeling with neighborhood sum degree topological indices to predict antibacterial drug properties. SVR outperformed linear regression, benefiting from feature selection and hyper-parameter tuning. Marshall and Forker (1982) investigated an ensemble learning approach for the analysis of mental disorder drugs. Using neighborhood degree-based indices derived from SMILES notations, the study identified optimal indices for predicting key physicochemical properties. Their findings showed the role of ensemble learning in better prediction accuracy, particularly for small datasets.

Additionally, it is important to note that none of the cited studies considered hydrogen atoms in their topological representations, which may neglect to important contribution to molecular properties. Furthermore, the all prior work relied on degree-based topological indices, which capture only local atomic environments. In our earlier study, the predictive power of topological indices for drug properties was explored using regression models along with distance-based indices. However, the influence of hydrogen configuration was not considered (Kour et al., 2024). In contrast, our present study demonstrated a novel comparison of explicit hydrogen and all hydrogen structures, using distance based indices that effectively capture molecular branching and spatial arrangement. Benchmarking LR and SVR, we observed that SVR provided superior accuracy for non-linear relationships, while LR performed well in strongly linear cases. This novel approach refines QSAR modeling, demonstrating how molecular representation influences predictive accuracy and optimizing regression techniques. The primary objective of this work is to understand the impact of hydrogen configuration on the prediction of six physicochemical properties using two regression techniques. This work aims to evaluate how different molecular representations impact prediction accuracy across multiple properties.

The major contributions in this study are:

• A comparative assessment of SVR and LR in handling linear and non-linear relationships.

• A detailed evaluation of six physicochemical properties using both molecular representations.

• The demonstration of how distance-based topological indices, combined with SVR, can enhance property prediction and serve as practical tool to accelerate early stage drug discovery.

## 2 Methodology and data collection

### 2.1 Drugs analysis

This study focuses on fifteen TCA drugs which have different molecular structure and clinical importance. Table 1 list their chemical structures and therapeutic uses, highlighting their role in treating depression and anxiety.

**TABLE 1:** TCA drugs with their chemical structure and therapeutic uses.

Drugs	Abbreviation	Chemical structures	Therapeutic uses
Alprazolam	ALP	C_17_ H_13_ Cl N_4_	Used to treat generalized anxiety disorder, panic disorder, and off-label for insomnia, premenstrual syndrome, and depression in adults
Amitriptyline	AMT	C_20_ H_23_ N	Used for major depressive disorder, neuro-pathic pain, chronic tension-type headache, migraine prophylaxis in adults, and nocturnal enuresis in children aged 6+ when other treatments fail
Amoxapine	AMX	C_17_ H_16_ Cl N_3_ O	For relieving depression symptoms in neurotic, reactive, endogenous, and psychotic depression, as well as depression associated with anxiety or agitation
Buspirone	BSP	C_21_ H_31_ N_5_ O_2_	Used to manage anxiety disorders or provide short-term relief from anxiety symptoms
Clomipramine	CLM	C_19_ H_23_ Cl N_2_	Used for obsessive-compulsive disorder, related conditions, and off-label for depression, chronic pain, narcolepsy, and autism
Desipramine	DSP	C_18_ H_22_ N_2_	Relieves symptoms of depressive syndromes, particularly endogenous depression, and manages chronic peripheral neuropathic pain, anxiety disorders, and ADHD (second or third-line treatment)
Desvenlafaxine	DVF	C_16_ H_25_ N O_2_	To treat major depressive disorder in adults and is also prescribed off-label for hot flashes in menopausal women
Diazepam	DZM	C_16_ H_13_ Cl N_2_ O	Used to treat anxiety, muscle spasms, acute alcohol withdrawal, spasticity, and as an adjunct for epilepsy, with indications for short-term anxiety relief, pre-surgical sedation in adults, and specific seizure episodes in children
Fluoxetine	FLX	C_17_ H_18_ F_3_ N O	Used for major depressive disorder, obsessive-compulsive disorder, bulimia nervosa, acute panic disorder, PMDD, and in combination with olanzapine for Bipolar I Disorder-related and treatment-resistant depression
Imipramine	IMP	C_19_ H_24_ N_2_	Used to relieve depression symptoms and reduce enuresis in children 6+, with off-label uses for panic disorders, ADHD, bulimia nervosa, bipolar depression, PTSD, and neuropathic pain
Lorazepam	LRZ	C_15_ H_10_ Cl_2_ N_2_ O_2_	Used for anxiety relief, sedation, and status epilepticus, with off-label uses for alcohol withdrawal, muscle spasms, insomnia, panic disorder, and more
Nortriptyline	NTP	C_19_ H_21_ N	Used to relieve symptoms of major depressive disorder (MDD) and off-label for chronic pain, myofascial pain, neuralgia, and irritable bowel syndrome
Oxazepam	OZP	C_15_ H_11_ Cl N_2_ O_2_	Used to manage anxiety disorders, provide short-term anxiety relief, and treat alcohol withdrawal symptoms
Protriptyline	PTP	C_19_ H_21_ N	Used for the treatment of major depression
Trimipramine	TMP	C_20_ H_26_ N_2_	Used to treat depression, including cases accompanied by anxiety, agitation, or sleep disturbances

**FIGURE 1:**
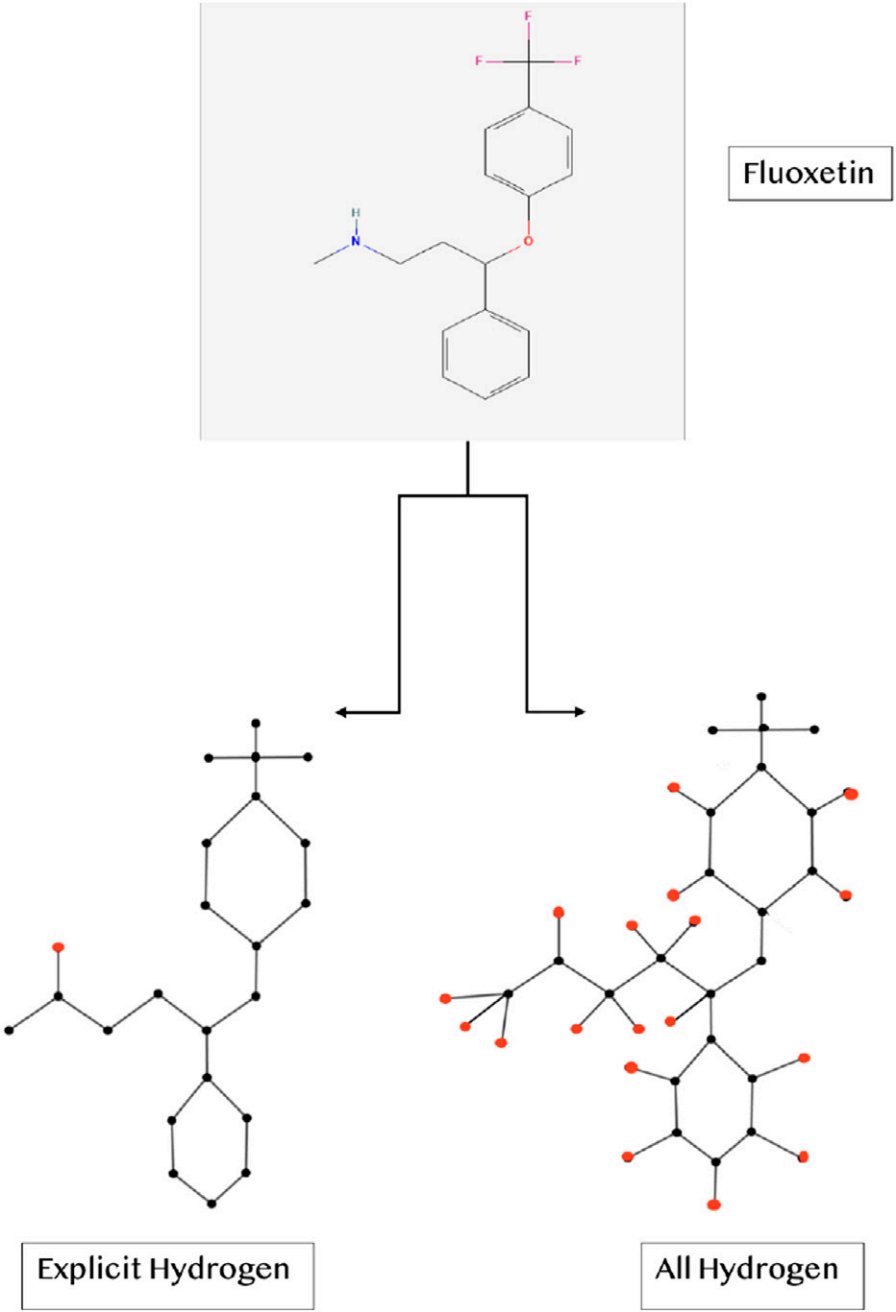
Two configurations of a molecule (Example: Fluoxetine).

Two molecular representations, one including explicit hydrogen only and the other including all the hydrogen, were analyzed to understand the influence of hydrogen on the properties of drugs. Figure 1 presents an example of Fluoxetine, showing its two different configurations-one with explicit hydrogen and other with all hydrogen. The hydrogen atoms are highlighted in red color. Six physicochemical properties listed in Table 2, were obtained from PubChem (2025) and ChemSpider (2025). These properties help us understand the thermodynamic and structural characteristics of these compounds in further computational analyses.

**TABLE 2:** Physicochemical properties of the TCA drugs.

Drugs	Boiling point (BP)	Enthalpy (E)	Flash point (FP)	Molar refractivity (MR)	Polarizability (P)	Molar volume (MV)
Alprazolam	509	77.9	261.6	88.2	35	225.6
Amitriptyline	398.2	64.9	174	91.5	36.3	257.8
Amoxapine	469.9	73.2	238	86.8	34.4	228.2
Buspirone	613.9	91.1	325.1	106.8	42.4	310.7
Clomipramine	434.2	69	216.4	93.8	37.2	281.2
Desipramine	407.4	65.9	160.5	84.2	33.4	254.3
Desvenlafaxine	403.8	69.1	193.2	77.8	30.9	236.1
Diazepam	497.4	76.5	254.6	80.9	32.1	225.9
Fluoxetine	395.1	64.5	192.8	79.9	31.7	266.7
Imipramine	403.1	65.4	179.7	88.9	35.3	269.2
Lorazepam	543.6	86.5	282.6	81	32.1	211.2
Nortriptyline	403.4	65.5	194.9	86.8	34.4	242.9
Oxazepam	516.6	83	266.2	76.4	30.3	201.9
Protriptyline	407.7	66	198.3	84.8	33.6	256.5
Trimipramine	411.8	66.4	183.3	93.5	37.1	286.1

### 2.2 Topological indices

The study explores the relationship between molecular properties and atomic arrangements using distance-based topological indices which are presented in Table 3. Similarly, calculations were performed for fifteen TCA drugs, analyzed with explicit hydrogen and with all the hydrogen. The results, presented in Tables 4, 5, provide numerical values that represent structural connectivity and molecular topology. These indices were selected due to their ability to capture spatial and str curtal complexity of the molecules. They effectively encode connectivity and branching patterns that influence molecular behavior.

**TABLE 3:** TCA drugs with their chemical structure and therapeutic uses.

Topological index	Notation	Formula
Wiener Index Wiener (1947)	W(G)	$W\left(G\right)=\sum_{1\leq a<b\leq n}d\left(y_{a},y_{b}\right)$
Hyper-Wiener Index Randić (1993)	WW(G)	$WW\left(G\right)=\sum_{1\leq a<b\leq n}\frac{d\left(y_{a},y_{b}\right)+d^{2}\left(y_{a},y_{b}\right)}{2}$
Harary Index Plavšić et al. (1993)	H(G)	$H\left(G\right)=\sum_{1\leq a<b\leq n}\frac{1}{d\left(y_{a},y_{b}\right)}$
Detour Index Lukovits (1996)	D(G)	$D\left(G\right)=\sum_{1\leq a<b\leq n}D\left(y_{a},y_{b}\right)$
Detour Harary Index Fang et al. (2018)	DH(G)	$DH\left(G\right)=\sum_{1\leq a<b\leq n}\frac{1}{D\left(y_{a},y_{b}\right)}$

**TABLE 4:** Topological indices of the drugs with explicit hydrogen.

Drugs	W	WW	H	D	DH
Alprazolam	926	2770	81.4698	2845	24.1903
Amitriptyline	882	2780	70.7087	2581	26.2091
Amoxapine	1075	3398	85.8329	3291	25.4936
Buspirone	2514	13028	102.7360	3764	57.3198
Clomipramine	995	3194	78.0429	2861	28.6732
Desipramine	882	2780	70.7087	2581	26.2091
Desvenlafaxine	897	2846	71.6540	1329	46.9409
Diazepam	726	2077	69.4905	1901	24.9069
Fluoxetine	1292	4946	77.8563	1772	54.1895
Imipramine	882	2780	70.7087	2581	26.2091
Lorazepam	1034	3076	86.5452	2601	33.9740
Nortriptyline	882	2780	70.7087	2581	26.2091
Oxazepam	928	2731	80.6476	2362	30.8960
Protriptyline	882	2780	70.7087	2581	26.2091
Trimipramine	979	3081	78.4611	2808	30.3429

**TABLE 5:** Topological indices of the drugs with all the hydrogen.

Drugs	W	WW	H	D	DH
Alprazolam	2936	10289	168.8604	7858	68.2119
Amitriptyline	5252	20371	239.9619	12117	126.6943
Amoxapine	3564	12697	193.5572	9689	78.4991
Buspirone	12709	69193	366.6186	18125	244.3614
Clomipramine	5462	21023	250.8310	12557	134.2503
Desipramine	4538	16736	226.4617	10943	114.0553
Desvenlafaxine	4836	17076	248.9564	7008	179.4176
Diazepam	2497	8380	154.1603	5741	71.3523
Fluoxetine	4433	17760	198.7027	6065	148.3364
Imipramine	5462	21023	250.8310	12557	134.2503
Lorazepam	2142	7027	139.4702	5038	62.4229
Nortriptyline	4346	16147	216.0927	10521	106.9993
Oxazepam	2142	7027	139.4702	5038	62.4229
Protriptyline	4267	15583	218.2697	10307	111.5984
Trimipramine	6278	24110	279.5762	14063	156.9178

Where, $d\left(y_{a},y_{b}\right)$ be the distance between the vertices $y_{a}$ and $y_{b}$ and $D\left(y_{a},y_{b}\right)$ be the length of the longest path between the vertices $y_{a}$ and $y_{b}$ .

The formulas for the topological indices remain the same for all the drugs, but they are calculated in two different ways according to the representation of hydrogen, one with only explicit hydrogen and other with all hydrogen. For preprocessing, all drug structure were standardized and convert into graph-based representations. These calculations are performed using Python and its libraries. RDKit is used to handle the molecular structures, NetworkX helps in creating the adjacency and distance matrices, and NumPy takes care of the numerical operations. The input for the molecular structures is provided in SMILES format—a simple text representation of molecules. First, molecules from PubChem are converted into graphs using RDKit. For explicit hydrogen calculation, the skeletal form of the molecule with Chem. MolFromSmiles (smiles) is used. And, for all hydrogen, we add explicit hydrogen atoms with Chem. AddHs(mol) which consider all the hydrogen present in a molecule.

### 2.3 Regression model

#### 2.3.1 QSPR model

QSPR is a computational model which is used to predict the physical, chemical, or biological properties of molecules based on their molecular structure. QSPR models establish a mathematical relationship between molecular descriptor (topological index) and a target property (Todeschini and Consonni, 2009).

The formulation of QSPR is represented in Equation 1.

$$a=f\left(y_{1},y_{2},\ldots ,y_{n}\right)$$ (1)

where, a represents the target property which is a dependent variable, $y_{1},y_{2},\ldots ,y_{n}$ represents the topological index, and f is the mathematical function.

#### 2.3.2 Linear regression

Linear regression is a method used to establish the relationship between a dependent variable and an independent variable by fitting a straight line (Zaid, 2015).

The equation is represented in Equation 2.

$$u=v+w_{1}x_{1}+w_{2}x_{2}+\cdots +w_{r}x_{r}+e$$ (2)

where u is the dependent variable, $x_{1},x_{2},\ldots ,x_{r}$ are the independent variables, $w_{1},w_{2},\ldots ,w_{r}$ are the regression coefficients, v is the intercept term, and e represents the random error.

The following LR model in Equation 3 is employed to construct a QSPR model.

$$u=v+w\left(t\right)$$ (3)

where, u represents target property (dependent variable), t represents topological index (independent variable), v represents intercept or constant of regression, and w represents regression coefficient.

#### 2.3.3 SVR theory

Support Vector Machines (SVM), introduced by Vapnik and others in 1995, are based on the structural risk minimization principle and statistical learning theory (Vapnik, 2013). SVM has been successfully applied to a wide range of classification and regression problems (Cai et al., 2003b; Cai et al., 2003a; Cortes and Vapnik, 1995; Smola and Schölkopf, 2004; Rao and Gopalakrishna, 2009). When used for regression, they are called support vector regression. Traditionally, QSPR models have relied on LR to predict compound properties because it is simple and interpretable. However, LR struggles with non-linear data and is sensitive to unusual data points. SVR addresses these limitations by effectively capturing non-linear patterns and showing more accurate and reliable predictions. These advantages make SVR a strong tool for combining with topological indices in QSPR studies (Awad and Khanna, 2015; Ardeshir et al., 2021; Baştanlar and Özuysal, 2013; Yang et al., 2005).

SVR focuses on developing a predictive model between given input features and their target values. Given a training dataset $A=\;{\left\{\left(y_{i},g_{i}\right)\right\}}_{i=1}^{m}$ , where each input $y_{i}\in R^{d}$ represents a feature vector with dimension d and $g_{i}\in R$ represents the corresponding target value, the goal is to determine a function f(y) that can accurately map the approximate value of y to g .

SVR creates a function that is linear in a transformed feature space but can model complex, non-linear relationships in the original input space. This is done by applying a non-linear transformation β(y) to map the input data into a higher-dimensional space where linear regression can be performed effectively.

The regression function is defined in Equation 4.

$$f\left(y\right)=W^{T}\beta \left(y\right)+q$$ (4)

where:

• $W\in R^{d}$ is the weight vector that defines the orientation of the regression hyperplane in the feature space,

• β(y) denotes a feature mapping function that projects the input x into a higher-dimensional space,

• q∈R serves as a bias term, shifting the hyperplane’s position accordingly.

The major goal of the SVR model is to find the weight vector W and bias q that minimize a combination of two components: a regularization term, which controls model complexity, and a loss function, which measures the prediction error. The SVR optimization minimizes the objective function Equation 5 subject to constraints Equations 6-8.

$$\underset{W,q,\zeta_{i},\zeta_{i}^{*}}{\min}\frac{1}{2}\|W{\|}^{2}+C\overset{m}{\underset{i=1}{\sum }}\left(\zeta_{i}+\zeta_{i}^{*}\right)$$ (5)

subject to:

$$g_{i}-\left(W^{⊤}\beta \left(y_{i}\right)+q\right)\leq \epsilon +\zeta_{i}$$ (6)

$$\left(W^{⊤}\beta \left(y_{i}\right)+q\right)-g_{i}\leq \epsilon +\zeta_{i}^{*}$$ (7)

$$\zeta_{i},\zeta_{i}^{*}\geq 0$$ (8)

where:

• The term $\|W{\|}^{2}$ serves as a regularization factor, aiding in managing the model’s complexity.,

• The parameter C>0 functions as a regularization parameter, regulating the balance between model complexity and the allowance for deviations beyond ϵ ,

• The value ϵ≥0 defines the epsilon-insensitive zone (epsilon-tube) where errors within this range are not penalized in the loss function,

• The slack variables $\zeta_{i}$ and $\zeta_{i}^{*}$ quantify the extent to which training samples fall outside the epsilon-tube, allowing the model to handle data points that do not fit perfectly within the margin.

In this study, radical basis function (RBF) has been implemented. The RBF kernel is a kernel that maps data to a higher dimensional space and is defined in Equation 9.

$$K\left(y_{i},y_{j}\right)=\exp\left(-\alpha \|y_{i}-y_{j}{\|}^{2}\right).$$ (9)

where, α is a parameter, which is equal to $\frac{1}{2\gamma^{2}}$ ( γ is the free parameter).

### 2.4 Performance evaluation

#### 2.4.1 Coefficient of determination $\left(R^{2}\right)$

In regression analysis, the most commonly used statistic to assess model performance is the coefficient of determination $R^{2}$ . It indicates how much of the variation in the response variable is explained by the model. The value of $R^{2}$ ranges from 0 to 1, where a higher $R^{2}$ signifies a better model fit (Cameron and Windmeijer, 1997).

The formula for calculating R-squared is defined in Equation 10.

$$R^{2}=1-\frac{\sum {\left(z_{i}-{\hat{z}}_{i}\right)}^{2}}{\sum {\left(z_{i}-\bar{z}\right)}^{2}}$$ (10)

where, $z_{i}$ represents the actual value of the dependent variable, ${\hat{z}}_{i}$ represents the predicted value from the regression models, and $\bar{z}$ represents the mean of actual values.

#### 2.4.2 Root mean squared error

The Root Mean Squared Error (RMSE) in the dataset is determined by taking the square root of the mean squared differences between the observed values and predicted values (Awad and Khanna, 2015; Sharma, 2005), given in Equation 11.

$$RMSE=\sqrt{\frac{1}{n}\overset{n}{\underset{i=1}{\sum }}{\left(z_{i}-{\hat{z}}_{i}\right)}^{2}}$$ (11)

where, n is the number of the observations, $z_{i}$ is the actual value, and $\hat{z_{i}}$ is the predicted value.

## 3 Results and discussion

In this study, a QSPR analysis of fifteen TCA drugs has been performed, to understand their physicochemical properties, which play an important role in examining their efficacy, stability, and thermodynamic behavior. The focus is on the correlations between molecular topological indices and physicochemical properties, and the impact of hydrogen atoms. SVR and LR models with explicit hydrogen and all-hydrogen were used to predict the properties based on topological indices and compared their performances to find the more accurate model.

### 3.1 Heatmap analysis

**FIGURE 2:**
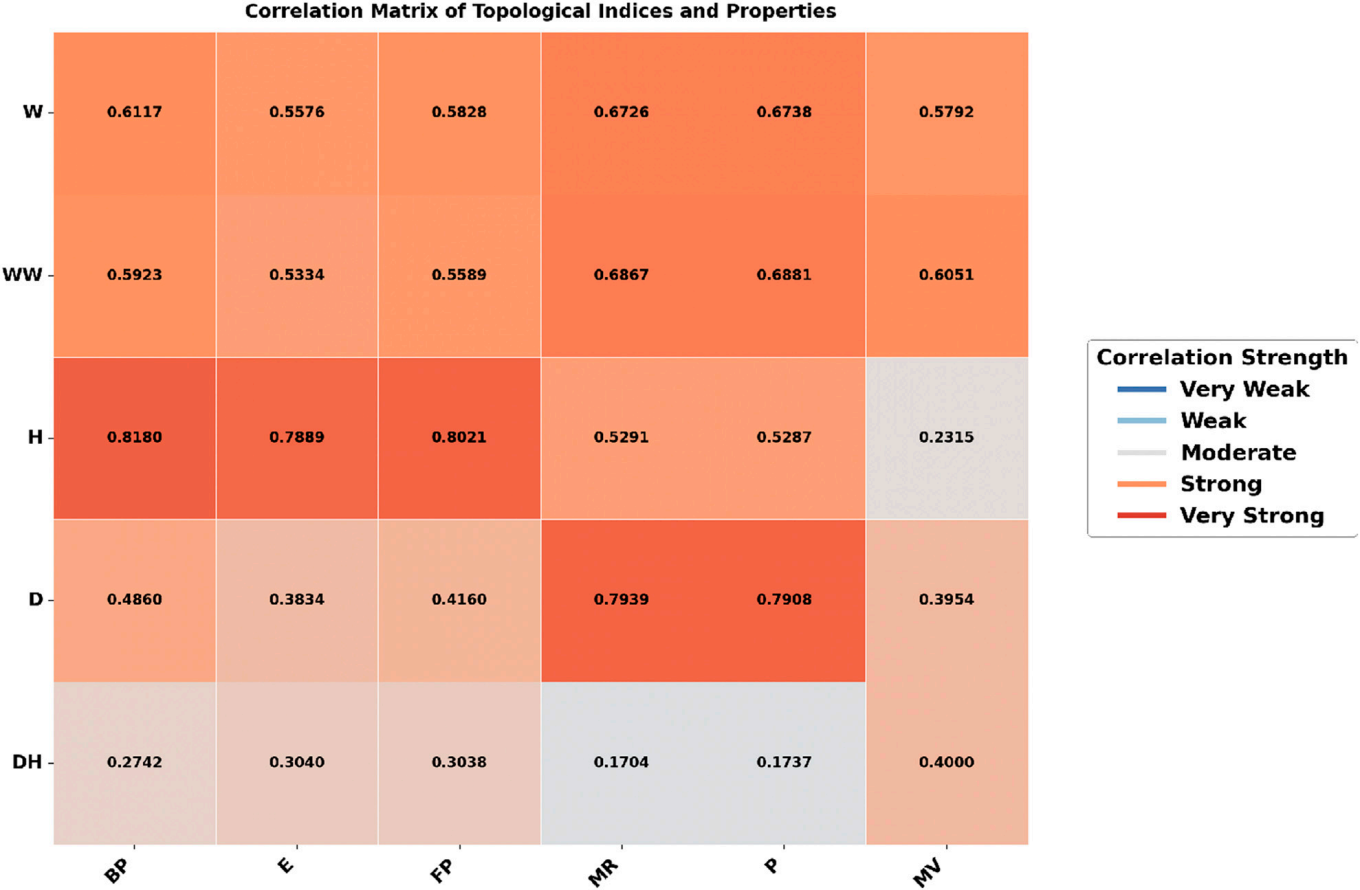
Correlation heatmap for TCA drugs with explicit hydrogen.

**FIGURE 3:**
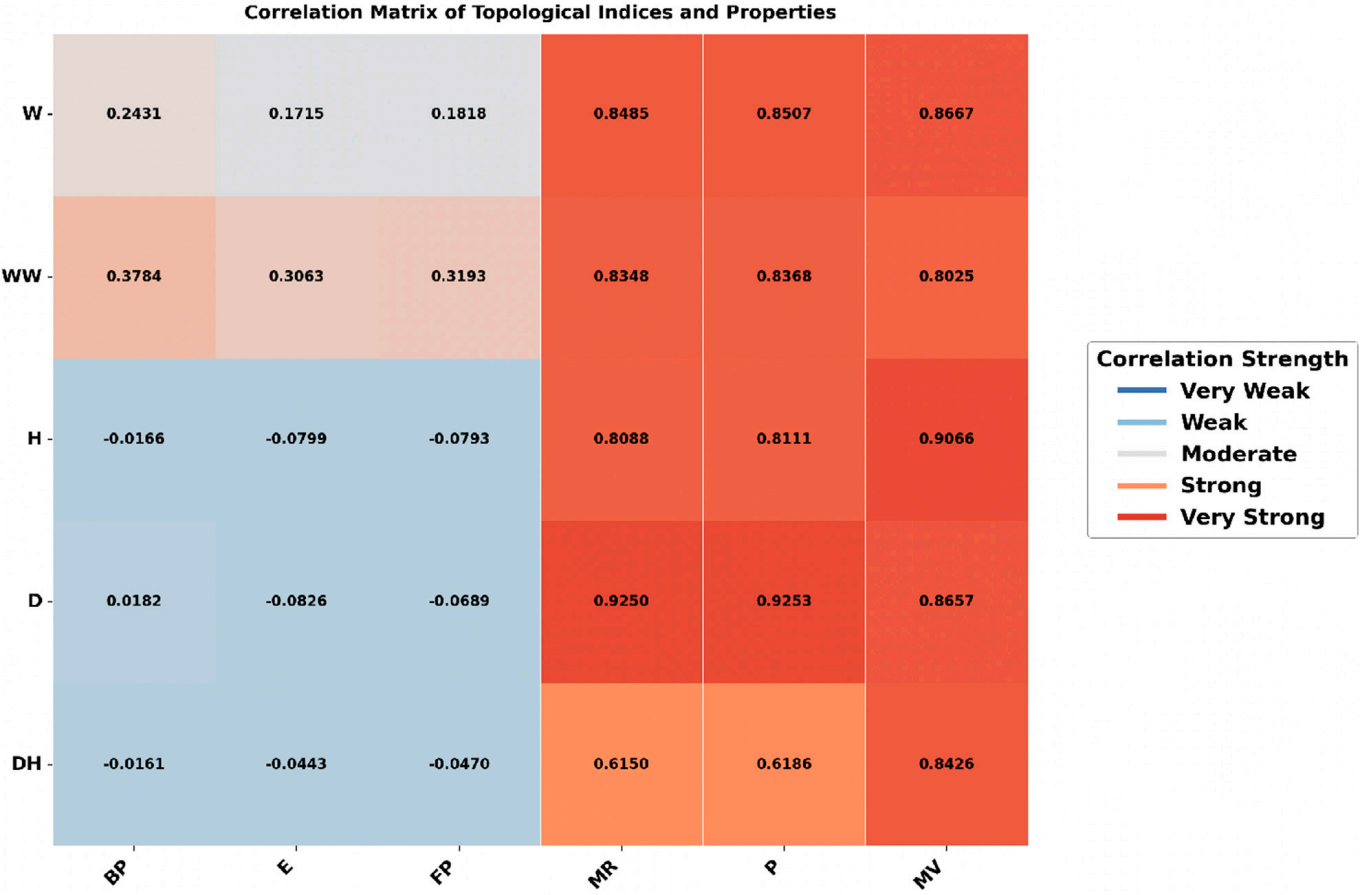
Correlation heatmap for TCA drugs with all the hydrogen.

The heatmap analysis, as shown in Figures 2, 3, compares the correlation between topological indices and physicochemical properties under two different molecular representations: explicit hydrogen and all hydrogen. The color intensity shows the strength of these relationships, darker colors mean a stronger correlation, while lighter colors mean a weaker one.

Figure 2, represents the dataset with explicit hydrogen only, shows a varied correlation pattern. The Harary Index has a strong correlation with Boiling Point (0.8180) and Flash Point (0.8021), but its correlation with Molar Refractivity (0.5291), Polarizability (0.5287), and Molar Volume (0.2315) is weaker. The Wiener and Hyper-Wiener indices show moderate relationships, especially with Molar Refractivity (0.6867) and Polarizability (0.6881). The Detour Harary Index has mostly weak correlations, as indicated by the lighter shades in the heatmap.

In contrast, Figure 3, where all hydrogen atoms are included, the correlation pattern appears more consistent. The Detour Index showed strong correlations for Molar Refractivity (0.9250), Polarizability (0.9253), and Molar Volume (0.8657). The Harary Index, which had strong correlations in Figure 1, now has much weaker correlations to Boiling Point (−0.0166) and Flash Point (−0.0793). The Detour Harary Index, which mostly has weak correlations, performs better with Molar Volume (0.8426) in this dataset.

### 3.2 SVR hyper-parameter tuning

The predictive model was developed using the SVR with the RBF kernel. The model was trained in Python using the scikit-learn library. The dataset was split into 80% training and 20% testing for better accuracy and validation. Hyper-parameter tuning was done to find the best values of the epsilon (ϵ) and cost (C) parameters, with epsilon ranging from 0.1 to 0.5 and C values set at 10, 50, 100, 500. The gamma parameter was adjusted to either “scale” or “auto” based on the requirement to achieve optimal results. To make the model more effective, 5-fold cross-validation was employed, where multiple SVR models were trained with different parameter settings. The best SVR model was trained using the optimal parameters and evaluated on the test dataset. The hyper-parameter tuning process was done separately for each physicochemical property, testing five different topological indices. The best index for each property was chosen based on the highest test $R^{2}$ value. This tuning process helped identify the best SVR models, leading to more accurate predictions and a stronger QSPR analysis. The final results, displayed in Table 6, show the best hyper-parameter values.

**TABLE 6:** Hyper-parameter tuning.

Property	With explicit hydrogen			With all hydrogen		
	C	ϵ	Gamma	C	ϵ	Gamma
Boiling Point	50	0.1	Auto	500	0.5	Scale
Enthalpy	10	0.5	Auto	100	0.1	Scale
Flash Point	50	0.5	Auto	500	0.2	Scale
Molar Refractivity	10	0.5	Auto	10	0.5	Auto
Polarizability	10	0.5	Auto	500	0.5	Auto
Molar Volume	50	0.5	Auto	50	0.1	Scale

### 3.3 Performance comparison: LR vs. SVR

**FIGURE 4:**
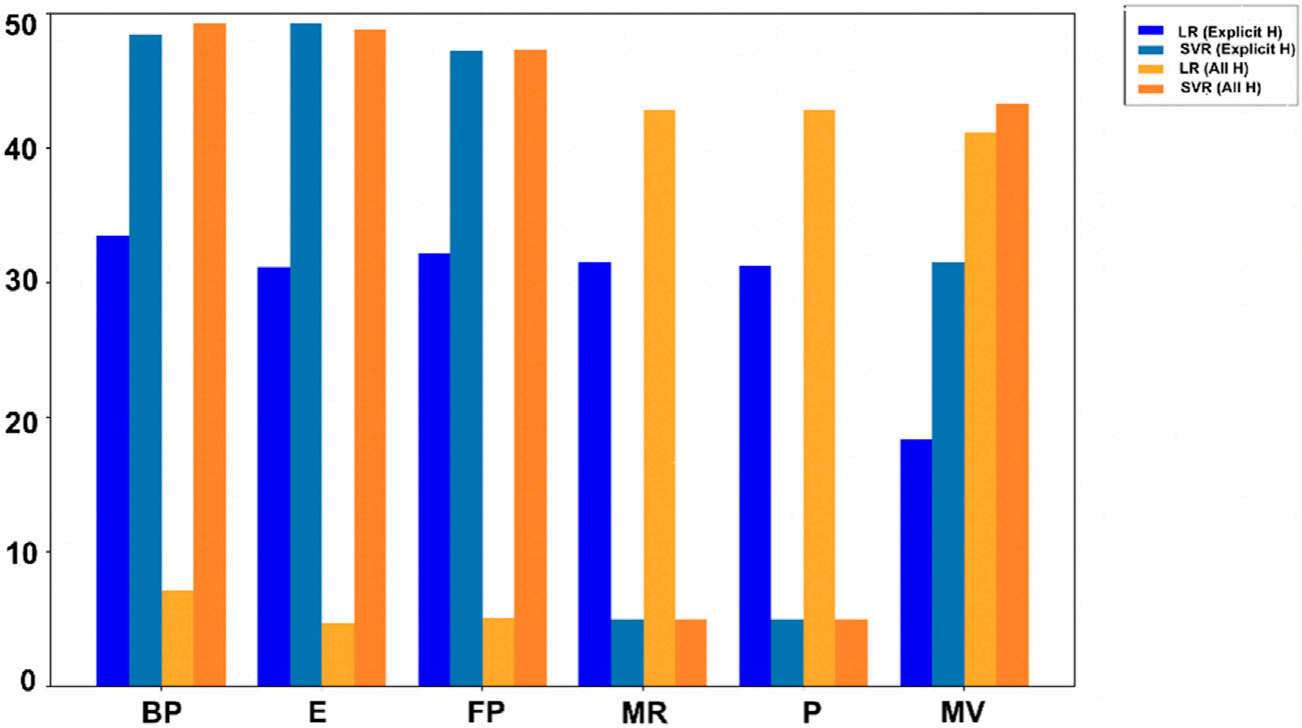
Comparison of $R^{2}$ values.

**FIGURE 5:**
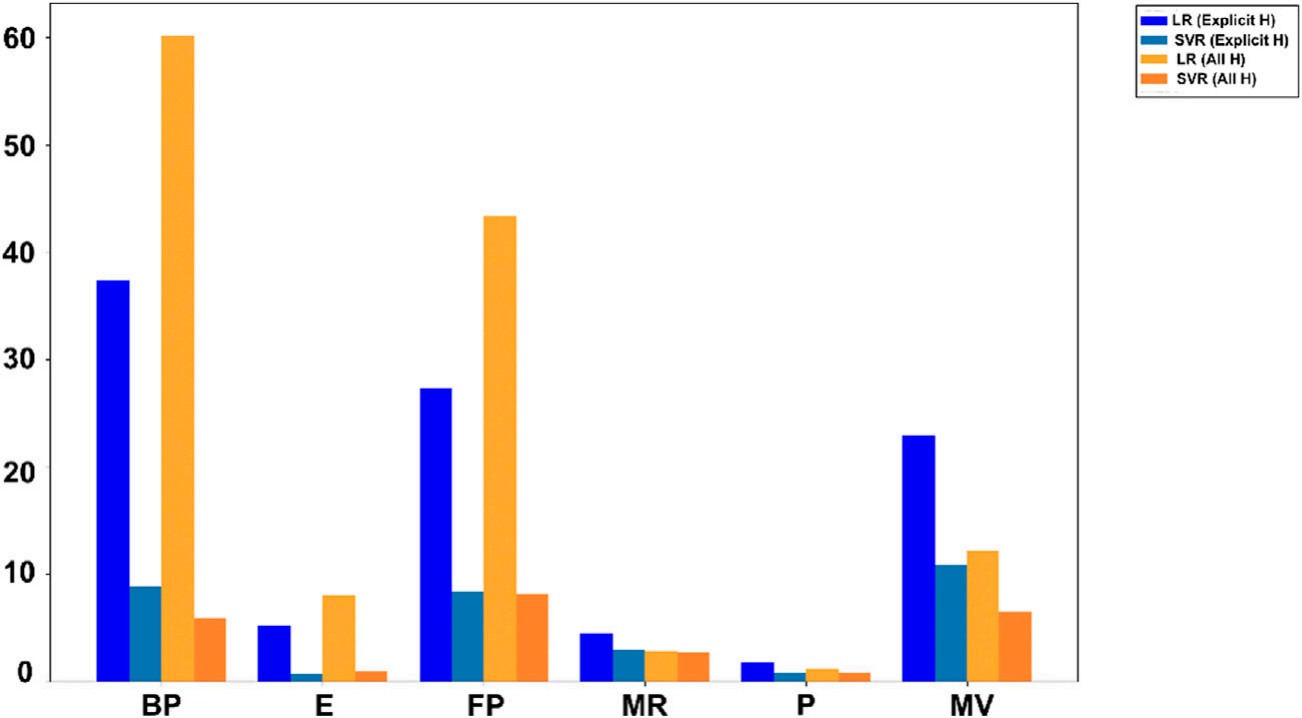
Comparison of RMSE values.

In Tables 7, 8, $R^{2}$ and RMSE values for LR and SVR models are presented for comparison. A higher $R^{2}$ indicates better accuracy and lower RMSE indicates few errors. Figures 4, 5 use bar graphs to visually compare these results. The findings suggest that SVR generally performs better, especially in all hydrogen model. However, LR showed better results for molar refractivity and polarizability, where it achieved much higher $R^{2}$ values despite SVR having slightly lower RMSEs but poor $R^{2}$ scores.

**TABLE 7:** Comparison for configuration with explicit hydrogen only.

Model	Property	Best TI	$R^{2}$	RMSE
SVR	Boiling Point	Harary Index	0.9681	8.8991
	Enthalpy	Harary Index	0.9853	0.7121
	Flash Point	Harary Index	0.9441	8.4131
	Molar Refractivity	Detour Index	0.1	2.9608
	Polarizability	Detour Index	0.1	0.8141
	Molar Volume	Harary Index	0.6308	10.8922
LR	Boiling Point	Harary Index	0.6692	37.3973
	Enthalpy	Harary Index	0.6223	5.1795
	Flash Point	Harary Index	0.6433	27.3783
	Molar Refractivity	Detour Index	0.6303	4.5457
	Polarizability	Detour Index	0.6253	1.8190
	Molar Volume	Hyper-Wiener Index	0.3662	22.9301

**TABLE 8:** Comparison for configuration with all hydrogen.

Model	Property	Best TI	$R^{2}$	RMSE
SVR	Boiling Point	Detour Harary Index	0.9861	5.8774
	Enthalpy	Detour Harary Index	0.9754	0.9197
	Flash Point	Detour Harary Index	0.9469	8.1953
	Molar Refractivity	Harary Index	0.1	2.7451
	Polarizability	Harary Index	0.1	0.8444
	Molar Volume	Detour Harary Index	0.8659	6.5639
LR	Boiling Point	Hyper-Wiener Index	0.1432	60.1869
	Enthalpy	Hyper-Wiener Index	0.0938	8.0226
	Flash Point	Hyper-Wiener Index	0.1019	43.4411
	Molar Refractivity	Detour Index	0.8557	2.8399
	Polarizability	Detour Index	0.8562	1.1269
	Molar Volume	Harary Index	0.8219	12.1539

### 3.4 Comparison of actual vs. predicted values

It is observed from the earlier results that SVR is better than LR in term of prediction. In this section, comparison of actual values of drug properties, along with the predicted values from the SVR model and LR model, using both the explicit hydrogen and all-hydrogen are presented. Overall, the predicted values follow the actual trends closely, demonstrating the strong predictive capability of SVR for most cases.

**FIGURE 6:**
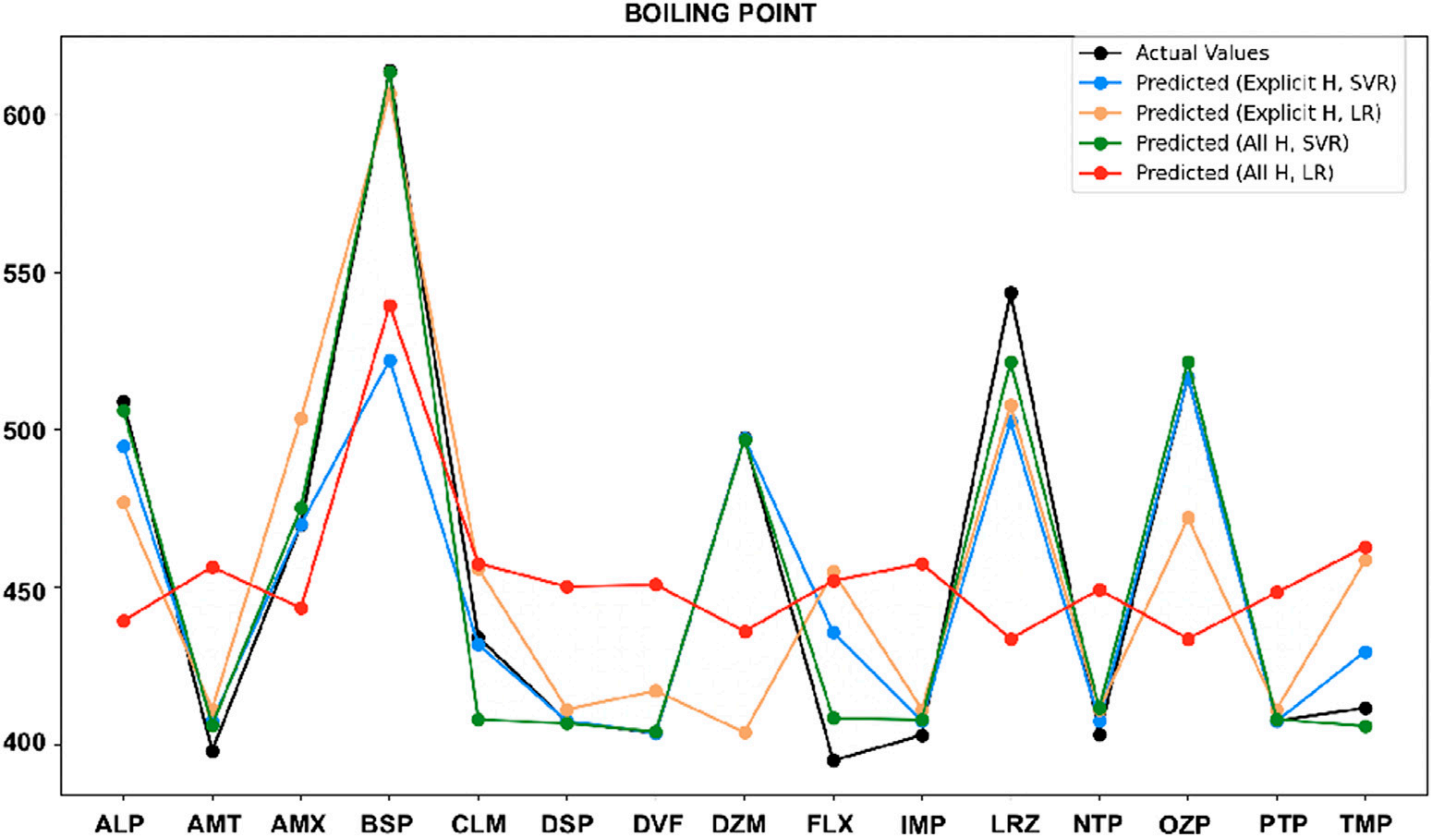
Actual vs. predicted values for boiling point.

**FIGURE 7:**
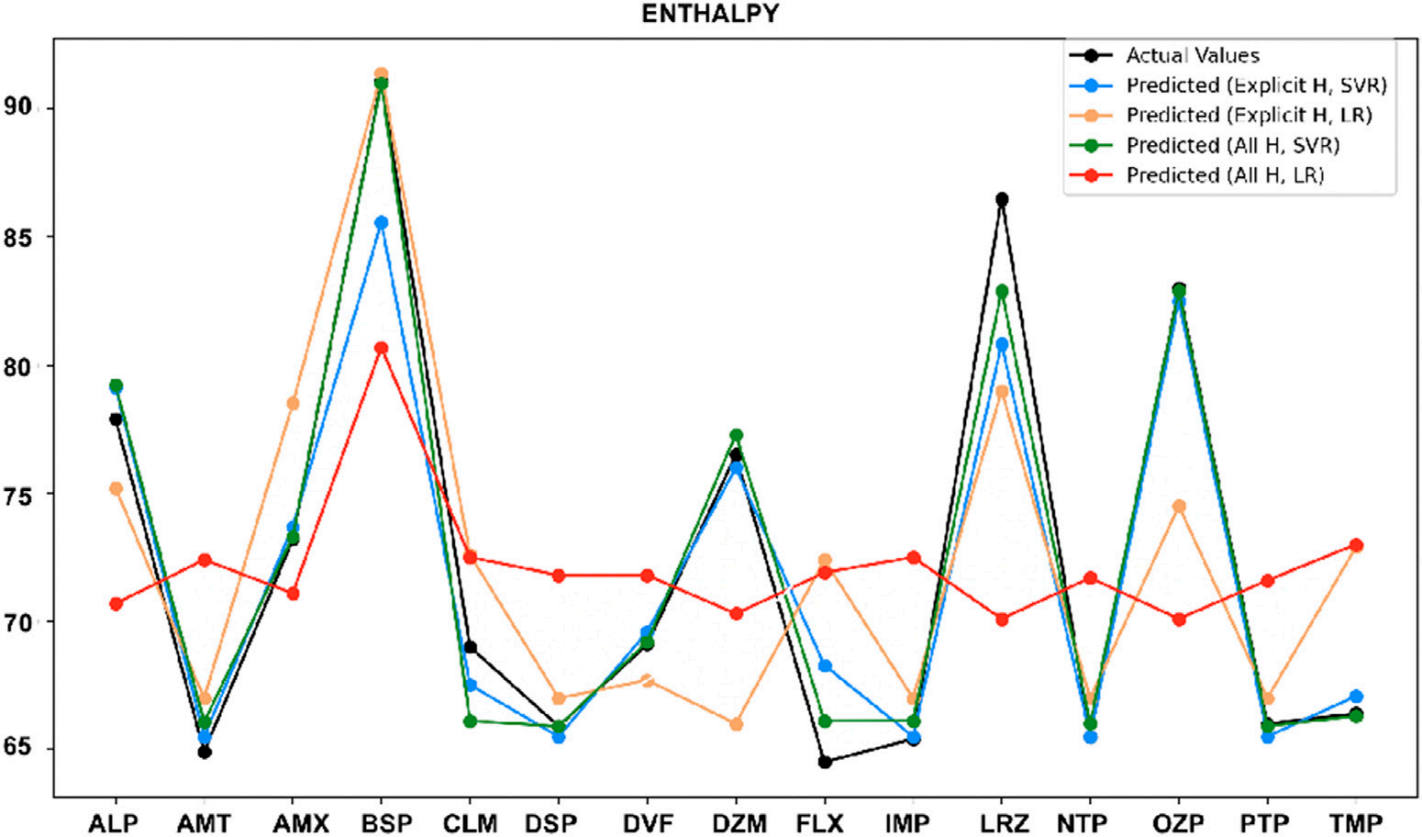
Actual vs. predicted values for enthalpy.

**FIGURE 8:**
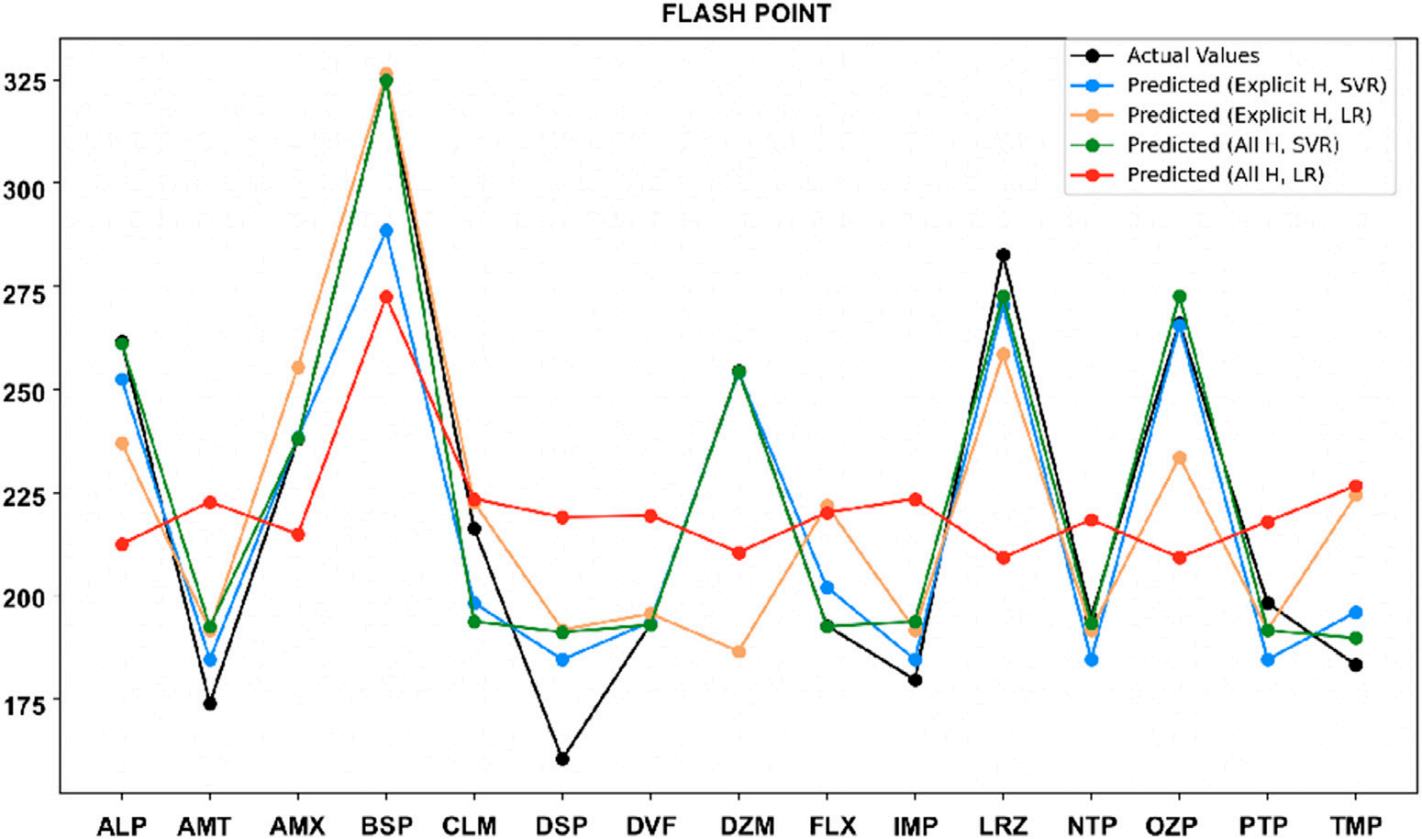
Actual vs. predicted values for flash point.

**FIGURE 9:**
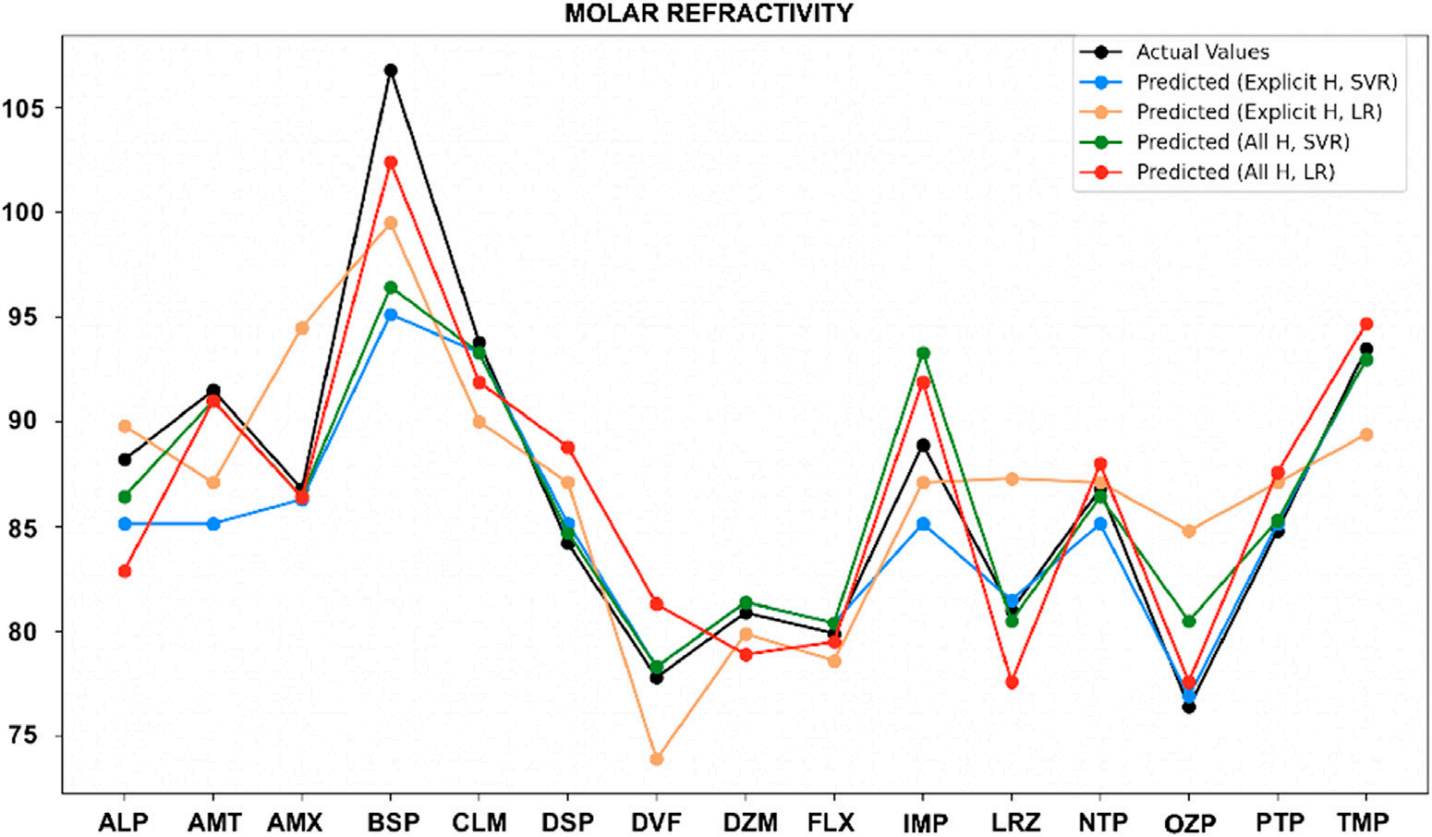
Actual vs. predicted values for molar refractivity.

**FIGURE 10:**
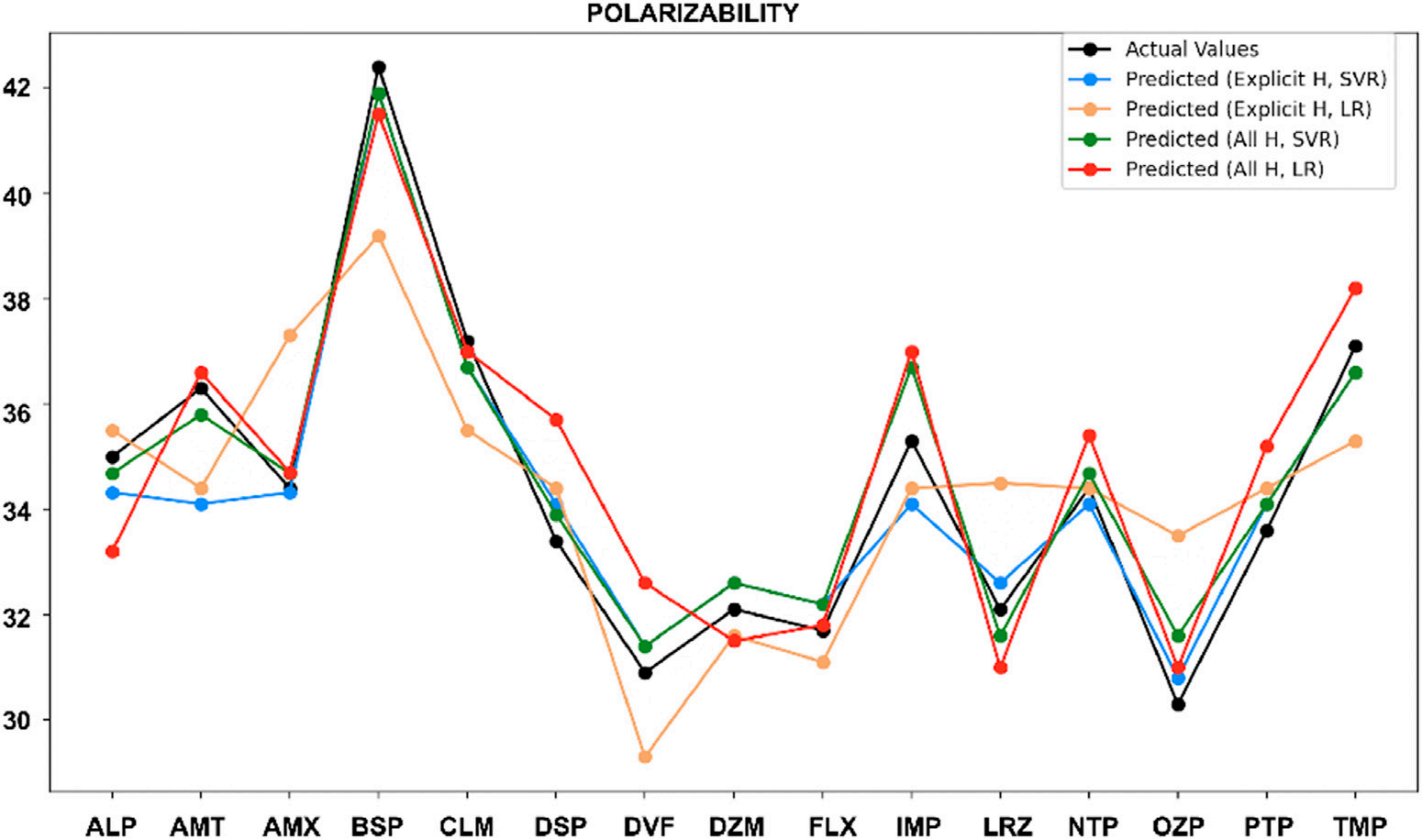
Actual vs. predicted values for polarizability.

**FIGURE 11:**
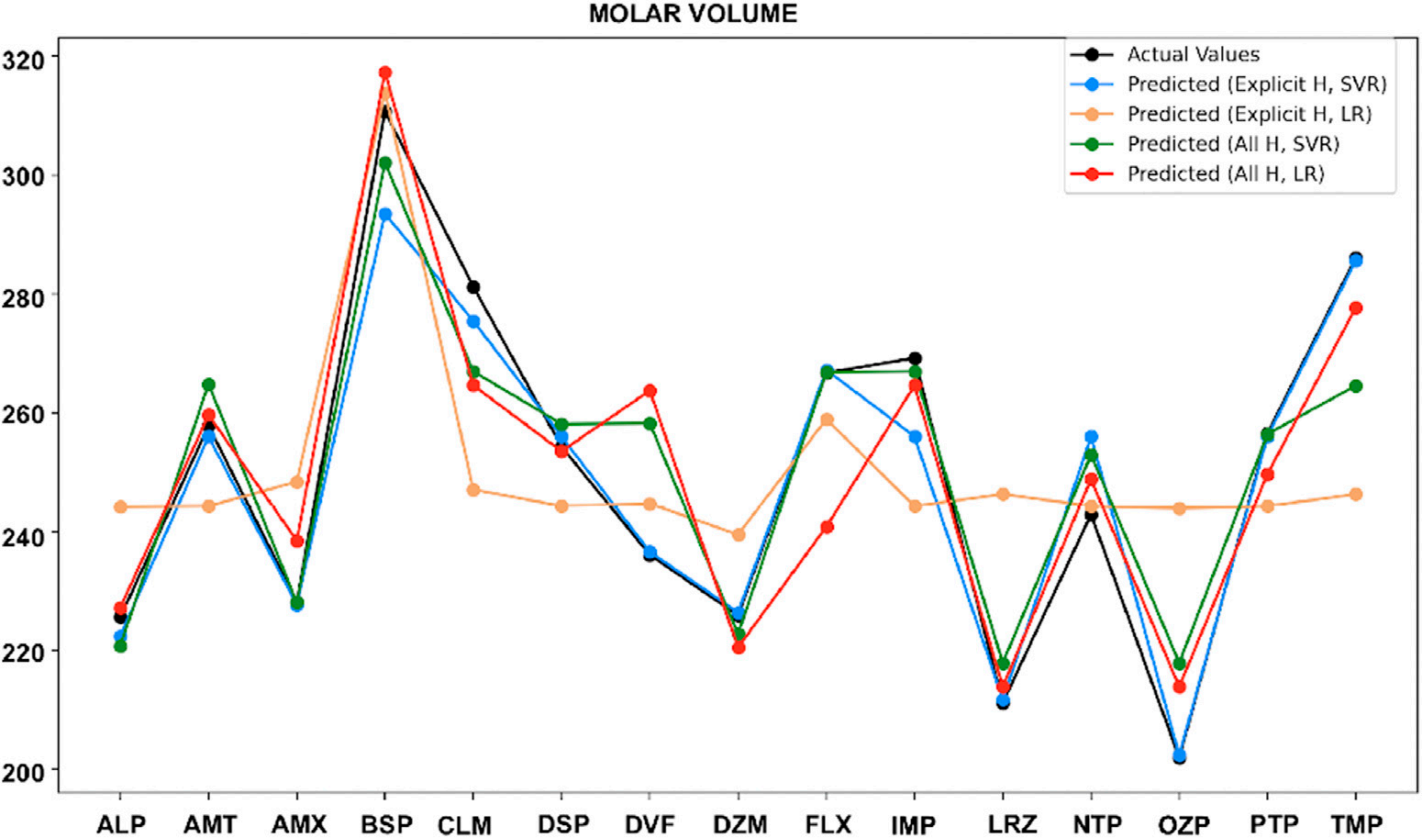
Actual vs. predicted values for molar volume.

• For boiling point (Figure 6), the all-hydrogen model with SVR closely matches actual values, especially for Amoxapine, Buspirone, Desipramine, Desvenlafaxine, and Diazepam, where the explicit hydrogen model shows large errors. For Clomipramine and Oxazepam, both explicit hydrogen and all hydrogen models with SVR perform equally. In the case of Nortriptyline, both SVR and LR work well but only with the explicit hydrogen model.

• For enthalpy (Figure 7), SVR with both explicit hydrogen and all hydrogen predicts accurately for most drugs, except Alprazolam, Clomipramine, Fluxoetine, and Lorazepam. However, for Buspirone, SVR with all hydrogen and LR with explicit hydrogen both models work similarly.

• For flash point (Figure 8), the all-hydrogen model with SVR performs best for most drugs, but the explicit hydrogen model is just as effective for Amoxapine and Oxazepam. The explicit hydrogen model with LR also performed well for few drugs like Buspirone, Desvenlafaxine and Nortriptyline.

• For molar refractivity (Figure 9), SVR with both explicit hydrogen and all hydrogen model performed well for most of the drugs, except Alprazolam, Buspirone and Imipramine. However, LR with all-hydrogen also showed good accuracy for Amitriptyline, Amoxapine, Fluoxetine, Oxazepam, while for Nortriptyline, LR worked well with both models.

• For polarizability (Figure 10), the all-hydrogen model provides good accuracy with SVR and LR, while the explicit hydrogen did not perform well with most of the drugs. However, for Amoxapine and Nortriptyline, the explicit hydrogen model still works well.

• For molar volume (Figure 11), SVR with both explicit and all-hydrogen models performs well, but the explicit hydrogen model is more accurate. LR with the all-hydrogen model also shows good performance for a few drugs.

These results show that molecular representation plays a key role in prediction accuracy. Both models perform well, but SVR with all-hydrogen model consistently provides more precise and accurate predictions, making it a better option for predicting drug properties. LR shows moderate performance, with occasional improvements when paired with explicit hydrogen models. This study underscores how including hydrogen in molecular structures enhances prediction accuracy.

It is evident that the SVR model generally outperformed the LR model, achieving higher $R^{2}$ and RMSE lower values, especially in capturing non-linear relationships through its use of kernel functions. This allows SVR to capture complex patterns in understanding the properties of the drugs. However, for certain properties, the LR model performs comparably or even better, suggesting linear correlations in those specific cases. SVR works well with small datasets, reducing the chance of overfitting while still giving strong predictions. Overall, because most of the data is non-linear and the drug structures vary, SVR is the better choice for predicting the physicochemical properties of the TCA drugs. However, LR can still be useful for properties that show a more linear relationship.

The findings of this study carry significant practical implications and offer a clear path for improving predictive modeling in drug discovery. It demonstrated the value of using machine learning techniques with chemically informative indices. Accurate prediction of properties is essential in the early stages of pharmaceutical development, where reliable estimation can guide compound selection. Such a framework can significantly reduce the need of time and cost experiment. Moreover, the ability to predict multiple molecular properties with high accuracy supports faster decision making in structure-activity relationship analysis. Overall, the proposed methodology offers a data-driven tool for better drug discovery pipeline by streamlining the evaluation of molecular drugs based on their predicted properties.

## 4 Conclusion

While SVR outperformed LR in most cases, LR also demonstrated strong performance for certain properties like molar refractivity and polarizability, making it valuable for understanding linear relationships between topological indices and specific molecular properties. These findings highlight the advantages of combining machine learning with topological indices for better drug property predictions and can guide future research and development of anti-depressant compounds. This study aims to predict the physicochemical properties of TCA drugs using a QSPR model that combines distance-based topological indices, SVR, and a traditional LR model. Two molecular configurations were analyzed: one with explicit hydrogen only and the other including all hydrogen. The results showed that including all hydrogen atoms led to stronger correlations, especially for properties like polarizability, molar refractivity, and molar volume. SVR outperformed LR in most of the cases, showing higher $R^{2}$ values and lower RMSE. This highlights that SVR is better at making predictions, notably when dealing with small-sized datasets. Hyper-parameter tuning played a key role in improving accuracy, making SVR a strong choice for predicting TCA drug properties.

In conclusion, adding all hydrogen atoms and using SVR has shown to be an effective approach for predicting the physicochemical properties of TCA drugs. It also helps in understanding the relationship between distance-based topological indices and molecular properties. While SVR outperformed LR in most cases, LR still worked well for some properties, such as molar refractivity and polarizability. This makes LR useful for understanding simple linear relationships between topological indices and specific molecular properties. These findings highlight the advantages of combining machine learning with topological indices for better drug property predictions and can guide future research and development of anti-depressants compounds. Future work could explore additional molecular configurations and different modeling techniques could to predictions more precise.

## Data Availability

All molecular structures, their properties, and their topological indices used in this study are provided as part of the main manuscript. Scripts and software for data analysis are available at the following repository: https://github.com/simran2410/tca_data.git. All necessary files and metadata are included to ensure reproducibility of the study.
